# Tissue‐type plasminogen activator (tPA) homozygous Tyr471His mutation associates with thromboembolic disease

**DOI:** 10.1002/mco2.392

**Published:** 2023-10-05

**Authors:** Yanyi Tao, Jiewen Ma, Yuanzheng Feng, Chenggang Gao, Tingting Wu, Yunqing Xia, Zhipeng Cheng, Yi Zhang, Tingting Liu, Yu Hu, Liang V. Tang

**Affiliations:** ^1^ Institute of Hematology Union Hospital Tongji Medical College Huazhong University of Science and Technology Wuhan China; ^2^ Department of Critical Care Medicine Union Hospital Tongji Medical College Huazhong University of Science and Technology Wuhan China

**Keywords:** arterial thrombosis, mutation, protease domain, tissue‐type plasminogen activator, venous thromboembolism

## Abstract

Tissue‐type plasminogen activator (tPA) encoded by *PLAT* is a major mediator that promotes fibrinolysis and prevents thrombosis. Pathogenetic mutations in *PLAT* associated with venous thromboembolism have rarely been reported. Here, we report the first case of a homozygous point mutation c.1411T>C (p.Y471H) in *PLAT* leading to thromboembolic events and conduct related functional studies. The corresponding tPA mutant protein (tPA‐Y471H) and wild‐type tPA (tPA‐WT) were synthesized in vitro, and mutant mice (*PLAT^H/H^* mice) were constructed. The molecular docking and surface plasmon resonance results indicated that the mutation impeded the hydrogen‐bonding interactions between the protease domain of tPA and the kringle 4 domain of plasminogen, and the binding affinity of tPA and plasminogen was significantly reduced with a difference of one order of magnitude. mRNA half‐life assay showed that the half‐life of tPA‐Y471H was shortened. The inferior vena cava thrombosis model showed that the rate of venous thrombosis in *PLAT^H/H^* mice was 80% compared with 53% in wild‐type mice. Our data suggested a novel role for the protease domain of tPA in efficient plasminogen activation, and demonstrated that this tPA mutation could reduce the fibrinolysis function of the body and lead to an increased propensity for thrombosis.

## Graphical abstract

1. We have identified the first *PLAT* homozygous mutation (p. Y471H) associated with thromboembolic disease, which is located in the protease domain of tPA and affects the effective activation of plasminogen.

2. Our study might provide a unique target for controlling plasmin generation, and also provides new insights and ideas for the diagnosis and treatment of patients with thrombosis in clinic.

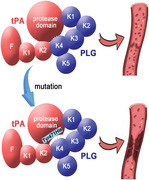

## 1 INTRODUCTION

Tissue‐type plasminogen activator (tPA) is a secreted serine protease of the trypsin family that catalyzes the critical step in fibrinolysis. It activates the circulating enzyme plasminogen, which can breakdown insoluble fibrin in the thrombus into soluble fibrin degradation products (FDPs).^1^ As thrombolytic agents, plasminogen activators are widely used to clear circulatory blockages caused by fibrin clots or thrombus. Recombinant tissue plasminogen activator (r‐tPA) has been approved by the US Food and Drug Administration as a clinical thrombolytic drug.^2^, ^3^

tPA is a single chained 70 kDa glycoprotein produced by endothelial, keratinocytes, and brain cells with low proteolytic activity and a very short circulation half‐life (2–6 min).^4^, ^5^ Under normal physiological conditions, the body maintains a balance between coagulation and fibrinolysis intravascularly by maintaining a balance between plasminogen activators (tPA/urokinase‐type plasminogen activator [uPA]) and inhibitors (plasminogen activator inhibitor‐1 [PAI‐1]/α2‐antiplasmin).^6^ Among plasminogen activators, tPA preferentially binds to plasminogen entrapped in fibrin compared with uPA. Because of this selectivity for plasminogen activation, tPA is more widely used clinically.^7^ PAI‐1, a member of the serine protease inhibitor (serpin) gene family, is a major physiologic inhibitor of tPA and uPA at the level of plasminogen activation, thereby inhibiting the plasminogen‐to‐plasmin conversion of plasminogen to plasmin.^8^, ^9^ α_2_‐Antiplasmin inhibits fibrinolysis mainly at the level of plasmin.^10^ If the balance of the coagulation and fibrinolysis is disrupted, fibrinolysis prevails, which will lead to the development of intravascular bleeding, whereas thrombosis follows when clotting prevails.

tPA is encoded by the *PLAT* (plasminogen activator, tissue) gene. Polymorphisms in *PLAT* (e.g., tPA‐7351C/T, tPA‐Alu insertion/deletion [I/D], tPA‐25 I/D) have been reported to be associated with clinical disorders such as stroke,^11^, ^12^, ^13^ myocardial infarction,^14^, ^15^ temporal lobe epilepsy (TLE),^16^ and increased susceptibility to some bacterial infections.^17^, ^18^ A study has reported that rare deleterious genetic variations in *PLAT* may be associated with prolonged illness duration and poorer prognosis in patients with early onset of Alzheimer's disease.^19^ Several earlier papers highlighted the association of familial defects in tPA release with venous thromboembolic events.^20^, ^21^ However, in the context of thrombophilia, pathogenetic mutations in *PLAT* have rarely been reported.

Here, we reported a newly identified rare homozygous point mutation c.1411T>C (p.Y471H) in the *PLAT* gene, which led to the mutation of amino acid 471 of tPA from tyrosine (Tyr) to histidine (His), and the proband carrying the mutation suffered from deep vein thrombosis. We investigated the effect of this mutation on tPA protein function, explored whether it was related to the patient's thromboembolic phenotype, and also explored the mechanism by which the mutation affected tPA.

## 2 RESULTS

### 2.1 The clinical characterization and genetic analysis of the probands

**FIGURE 1:**
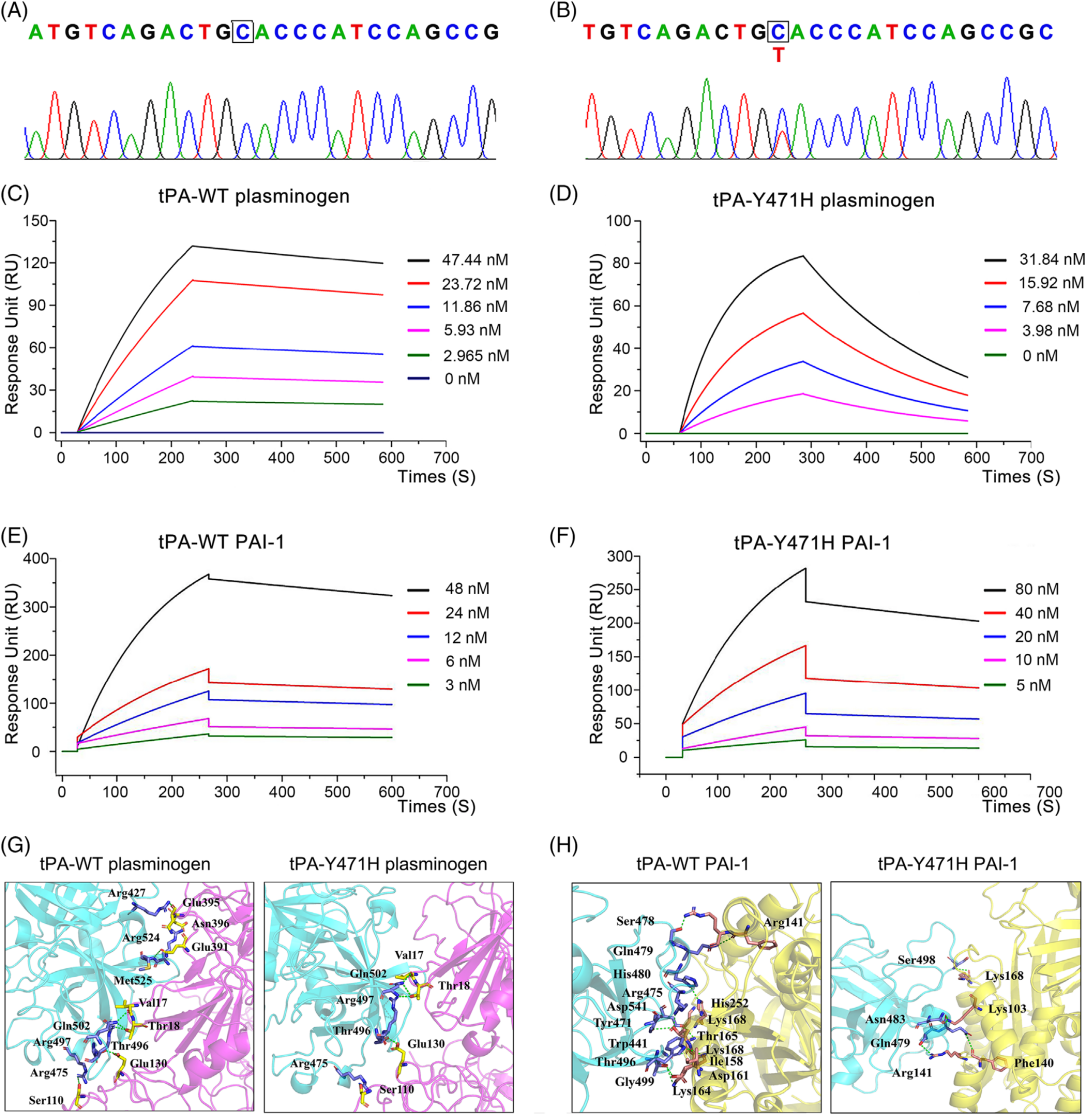
Genetic analysis of the probands, surface plasmon resonance (SPR), and molecular docking analysis of tissue‐type plasminogen activator (tPA) binding to plasminogen and plasminogen activator inhibitor‐1 (PAI‐1). (A) Gene sequencing results showed that the proband had a homozygous mutation of T base to C base at site 1411 of gene *PLAT*. (B) His parents and daughter were heterozygous for c.1411T>C. (C and D) SPR analysis of wild‐type tissue plasminogen activator (tPA‐WT) and tissue plasminogen activator with a p.Tyr471His (tPA‐Y471H) binding with plasminogen. (E and F) SPR analysis of tPA‐WT and tPA‐Y471H binding with PAI‐1. (G) Models of tPA‐WT (left panel) and tPA‐Y471H (right panel) bound to plasminogen, respectively. (H) Models of tPA‐WT (left panel) and tPA‐Y471H (right panel) bound to PAI‐1. Blue indicates tPA, purple indicates plasminogen, yellow indicates PAI‐1, and green lines indicates hydrogen bonds. The interacting nucleotides are presented in Table 1.

The proband was a male who first developed deep vein thrombosis of the left lower extremity without obvious inducement at the age of 29 years. As advised, the proband should take oral warfarin long after discharge to prevent thrombosis, but he stopped medication spontaneously after 1 month and was readmitted to the hospital for posterior tibial vein thrombosis of the right lower extremity nearly a year after self‐withdrawal. We performed genetic sequencing of peripheral blood from the proband and saliva samples from his immediate family members and measured the expression of tPA in the proband's plasma. Gene sequencing revealed that the proband had a homozygous c.1411T>C (p.Y471H) mutation of *PLAT* (Figure 1A), without common severe thrombophilias such as protein C, protein S, or antithrombin deficiency. The proband's parents and daughter were heterozygous carriers of this mutation (Figure 1B), but they did not develop any thromboembolic disease. The plasma tPA antigen level and enzyme activity of the proband were 3.42 ng/mL (reference, 4.67 ± 0.45 ng/mL) and 51.88 IU/mL (reference, 94.61 ± 7.4 IU/mL), respectively. The results suggested that the concentration and activity of plasma tPA were reduced in the proband carrying the homozygous point mutation.

### 2.2 Impact of tPA mutation on binding to plasminogen and PAI‐1 determined by SPR

We assessed the effect of tPA‐Y471H mutation on its binding to plasminogen and PAI‐1 by surface plasmon resonance (SPR). For the affinity constant (*K*_D_), compared with wild‐type tPA (tPA‐WT), the affinity of tPA‐Y471H binding with plasminogen was significantly decreased (2.22e‐9 ± 1.50e‐11 vs. 1.48e‐8 ± 1.13e‐9) (Figure 1C,D), and the affinity of tPA‐Y471H binding with PAI‐1 was slightly decreased (2.09e‐9 ± 1.42e‐9 vs. 5.64e‐9 ± 3.57e‐10) (Figure 1E,F). That is, the tPA‐Y471H variant displayed a less stable structure with them two compared with tPA‐WT.

### 2.3 Molecular docking of tPA and plasminogen or PAI‐1

To explore how the mutation affects the interaction between proteins. We modeled the binding of tPA protease domain to PAI‐1 and plasminogen. Compared with tPA‐WT, the tPA‐Y471H variant displayed a less stable structure binding to PAI‐1 and plasminogen (Figure 1G,H). PAI‐1 and plasminogen reported docking scores of −223.6 and −193.1, whereas tPA‐Y471H showed docking scores of −185.1 and −156.6 with tPA protease domain through hydrogen‐bonding interactions (Table 1). According to the docking results, we found that the amino acids in kringle 1 domain (Val17/Thr18, Thr18, Glu130, and Ser110) and kringle 4 domain (Asn396, Glu395, and Glu391) of plasminogen can bind to the amino acids in tPA‐WT protease domain (Arg427, Arg524, Met525, Gln502, Arg 497, Thr496, and Agr475) through hydrogen‐bonding interactions. However, the tPA‐Y471H protease domain only bound to the kringle 1 domain of plasminogen, as shown in Table 1. These results suggested that the mutation affected the binding of tPA protease domain to kringle 4 domain of plasminogen, and this might play a very important role in the binding of tPA to plasminogen.

**TABLE 1:** The interactions for both plasminogen and plasminogen activator inhibitor‐1 (PAI‐1) bound to tissue‐type plasminogen activator (tPA).

	tPA‐WT–plasminogen	tPA‐Y471H–plasminogen	tPA‐WT–PAI‐1	tPA‐Y471H–PAI‐1
Hydrogen bonds (nucleotides)	Arg427–Asn396, Arg524–Glu395, Met525–Glu391, Glu502–Val17/Thr18, Arg497–Thr18, Thr496–Glu130, Arg475–Ser110	Glu502–Val17/Thr18, Arg497–Thr18, Thr496–Glu130, Arg475–Ser110	Ser478/Glu479–Arg141, His480–His252, Arg475–Thr165, Asp541–Lys168, Tyr471–Thr165, Trp441–Ile158, Thr496–Asp161/Lys164, Gly499–Lys168	Ser498–Lys168, Asn483–Lys103, Gln479–Arg141/Phe140/Lys103
Docking scores (kcal/mol)	−193.1	−156.6	−223.6	−185.1

Abbreviations: Arg, arginine; Asn, asparagine; Asp, asparticacid; Gln, glutarnine; Glu, glutamicacid; Gly, glycine; His, histidine; Ile, isoleucine; Lys, lysine; Met, methionine; Phe, phenylalanine; Ser, serine; Thr, tyrosine; tPA‐WT, wild‐type tissue plasminogen activator; tPA‐Y471H, tissue plasminogen activator with p.Tyr471His; Trp, tryptophan; Tyr, tyrosine; Val, valine.

### 2.4 Effect of mutation on tPA‐promoted plasmin generation

**FIGURE 2:**
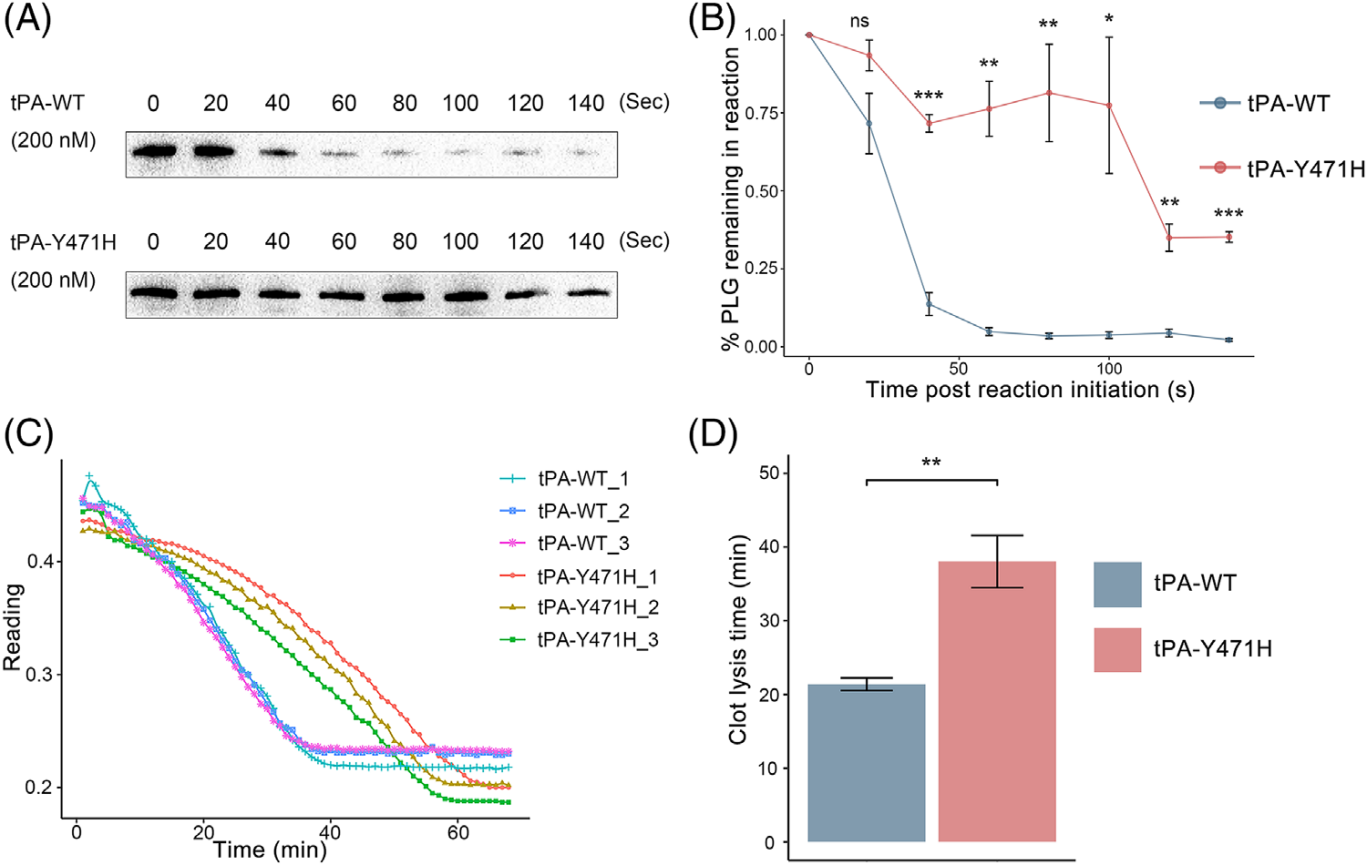
Tissue‐type plasminogen activator (tPA)‐mediated conversion of plasminogen to plasmin and clot lysis assay. (A) Time course of conversion of plasminogen to plasmin in the presence of fibrin detected by SDS‐PAGE and immunoblotting using anti‐plasminogen antibody. (B) Densitometry analysis of bands in (A) representing remaining percentage of zymogen plasminogen in sample (*n* = 3). The means ± standard deviation (SD) (*y*‐axis) are shown over time (*x*‐axis). ^*^, ^**^, ^***^, and ns on time denote differences in tissue plasminogen activator with a p.Tyr471His (tPA‐Y471H) from wild‐type tissue plasminogen activator (tPA‐WT). ^*^ *p* < 0.05; ^**^ *p* < 0.01; ^***^ *p* < 0.001; ns: not significant as determined by unpaired *t*‐test. (C) Clot lysis time of each group was calculated. ^**^ *p* < 0.01, unpaired *t*‐test. (D) Clot lysis curves monitored by turbidity measurement at 405 nm. Three independent experiments were performed in each group.

tPA was used to mediate plasminogen cleavage to plasmin in the presence of fibrin and the rate of plasminogen conversion was detected with densitometry measurements of Western‐blot analyses (Figure 2A,B). At 140 s, 97.79 ± 0.69% of total plasminogen catalyzed by tPA‐WT was converted to plasmin compared with 64.77 ± 2.97% of plasminogen catalyzed by tPA‐Y471H (*p* < 0.0001, *n* = 3). We also performed an experiment of tPA mediating fibrinolysis in thrombin‐induced plasma clot and the clot lysis time was determined by turbidity change (Figure 2C). The results showed that the process of clot lysis induced by tPA‐Y471H was significantly prolonged compared with that induced by tPA‐WT (21.41 ± 0.86 min vs. 38.02 ± 3.54 min, *p* = 0.0014), indicating that the process of fibrinolysis induced by the tPA variant was changed (Figure 2D).

### 2.5 Mutation reduced the fibrinolytic function of tPA

**FIGURE 3:**
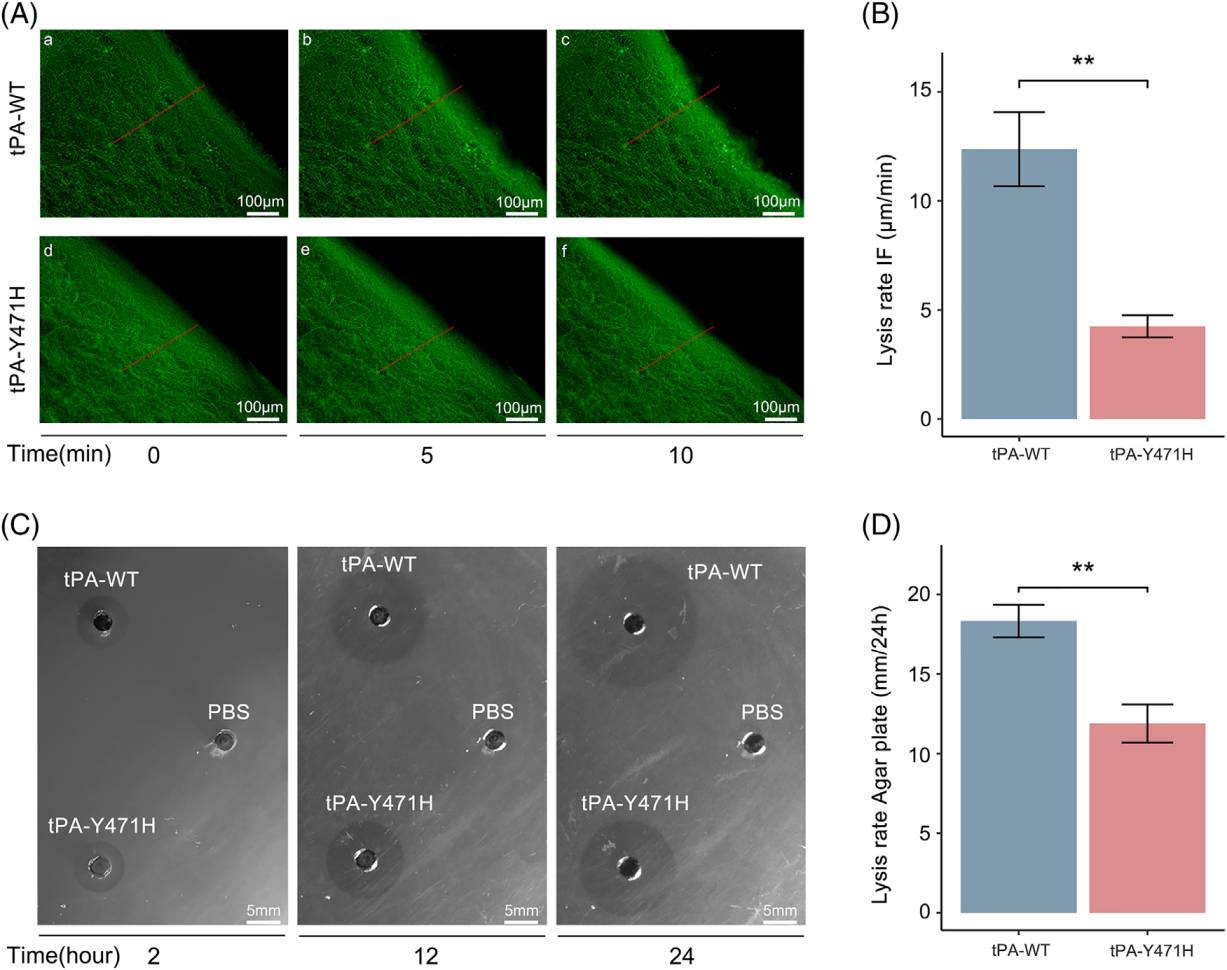
Mutation reduced the fibrinolytic function of tissue‐type plasminogen activator (tPA). (A) Clots were prepared with fibrinogen containing Alexa Fluor 594‐label and the fluid/fibrin interface was monitored by microscope using fluorescent tracing. Progression of the lysis front was observed after 0 min (a and c), 5 min (b and e), and 10 min (c and f). Scale bar, 100 μm. (B) The mean overall lysis rate was calculated. The graph bars represent mean ± standard deviation (SD) of three independent experiments. Unpaired *t*‐test was used for statistical analysis. ^*^ *p* < 0.05; ^**^ *p* < 0.01; ^***^ *p* < 0.001; ns: not significant. (C) Clot lysis assay by agar plate method. The panel was observed after 2, 12, and 24 h of incubation at 37°C. The three wells were wild‐type tissue plasminogen activator (tPA‐WT), tissue plasminogen activator with p.Tyr471His (tPA‐Y471H), and phosphate‐buffered saline (PBS). Scale bar, 5 mm. (D) Changes in diameter were observed and the mean lysis rate was calculated. The graph bars represent mean ± SD of three independent experiments, unpaired *t*‐test. ^*^ *p* < 0.05; ^**^ *p* < 0.01; ^***^ *p* < 0.001; ns: not significant.

Immunofluorescence imaging and agar plate assays were used to further verify the effect of the mutation on the fibrinolytic properties of tPA. tPA‐Y471H‐induced lysis of fibrin was significantly slowed down compared with tPA‐WT (12.37 ± 1.70 μm/min vs. 4.26 ± 0.51 μm/min, *p* = 0.0014) (Figure 3A,B). For agar plate assay, the disruption of the fibrin plate was visually monitored through a clear zone created by the enzymatic action of tPA (Figure 3C). The size of the clear zone created by tPA‐Y471H was significantly smaller than that created by tPA‐WT (18.33 ± 1.02 mm/24 h vs. 11.89 ± 1.20 mm/24 h, *p* = 0.0021) (Figure 3D). These data suggested that the mutation reduced the fibrinolytic function of tPA.

### 2.6 Fibrinolytic factors of the mutation *PLAT^H/H^* mice

**FIGURE 4:**
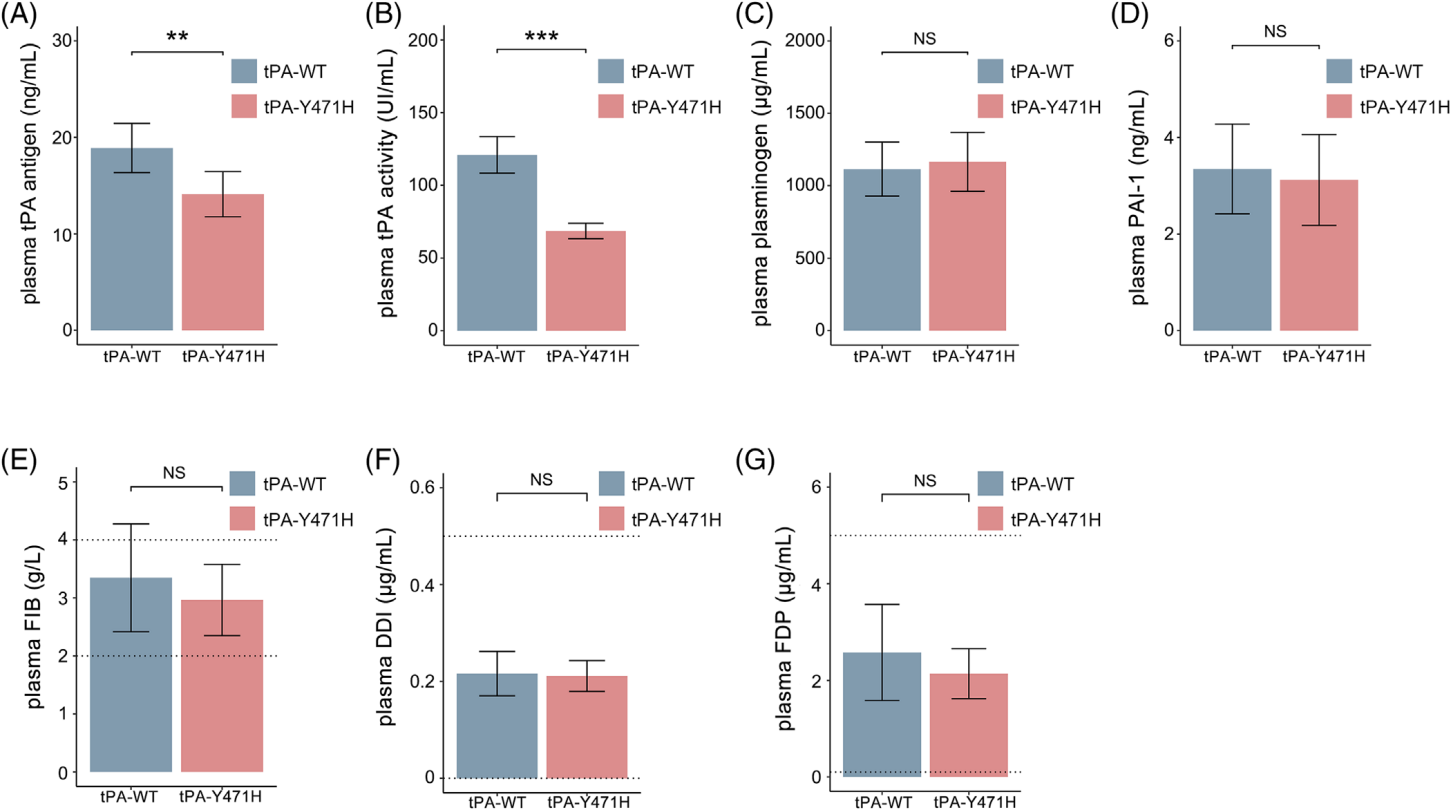
Changes in systemic fibrinolytic activity in *PLAT^H/H^* mice. (A) Plasma tissue‐type plasminogen activator (tPA) concentration by ELISA. (B) Plasma tPA activity by enzymatic assay. (C and D) Plasma plasminogen and plasminogen activator inhibitor‐1 (PAI‐1) concentrations by ELISA. (E and G) Plasma fibrinogen (FIB), D‐dimer (DDI), and fibrin degradation products (FDP) were measured by an automatic coagulator. Blue indicates wild‐type mouse group and red indicates *PLAT^H/H^* mouse group. *N* = 8 mice per group. Bars represent the group mean ± standard deviation (SD). Between the two dashed lines are the reference ranges for measurements from healthy control. An unpaired *t*‐test was used for statistical analysis. ^*^ *p* < 0.05; ^**^ *p* < 0.01; ^***^ *p* < 0.001; ns: not significant.

To evaluate the effect of the mutation on tPA and other related fibrinolytic factors, we detected total antigen level and enzymatic activity of tPA in the plasma and observed significant decrease in both end points in *PLAT^H/H^* mice compared with the control wild‐type mice (Figure 4 and Table S1). This mutation resulted in a decrease in tPA antigen level and enzymatic activity in *PLAT^H/H^* mice to 74.7% and 56.8% of normal levels, respectively, which was consistent with the clinical data of proband (Figure 4A,B). Plasma plasminogen antigen level and PAI‐1 antigen level did not differ significantly between the two groups (Figure 4C,D). Also, there were no obvious differences in the plasma of fibrinogen (FIB), D‐dimer (DDI), and FDPs between *PLAT^H/H^* mice and wild‐type mice (Figure 4E–G).

### 2.7 Mutation increases the propensity for thrombosis in *PLAT^H/H^* mice

**FIGURE 5:**
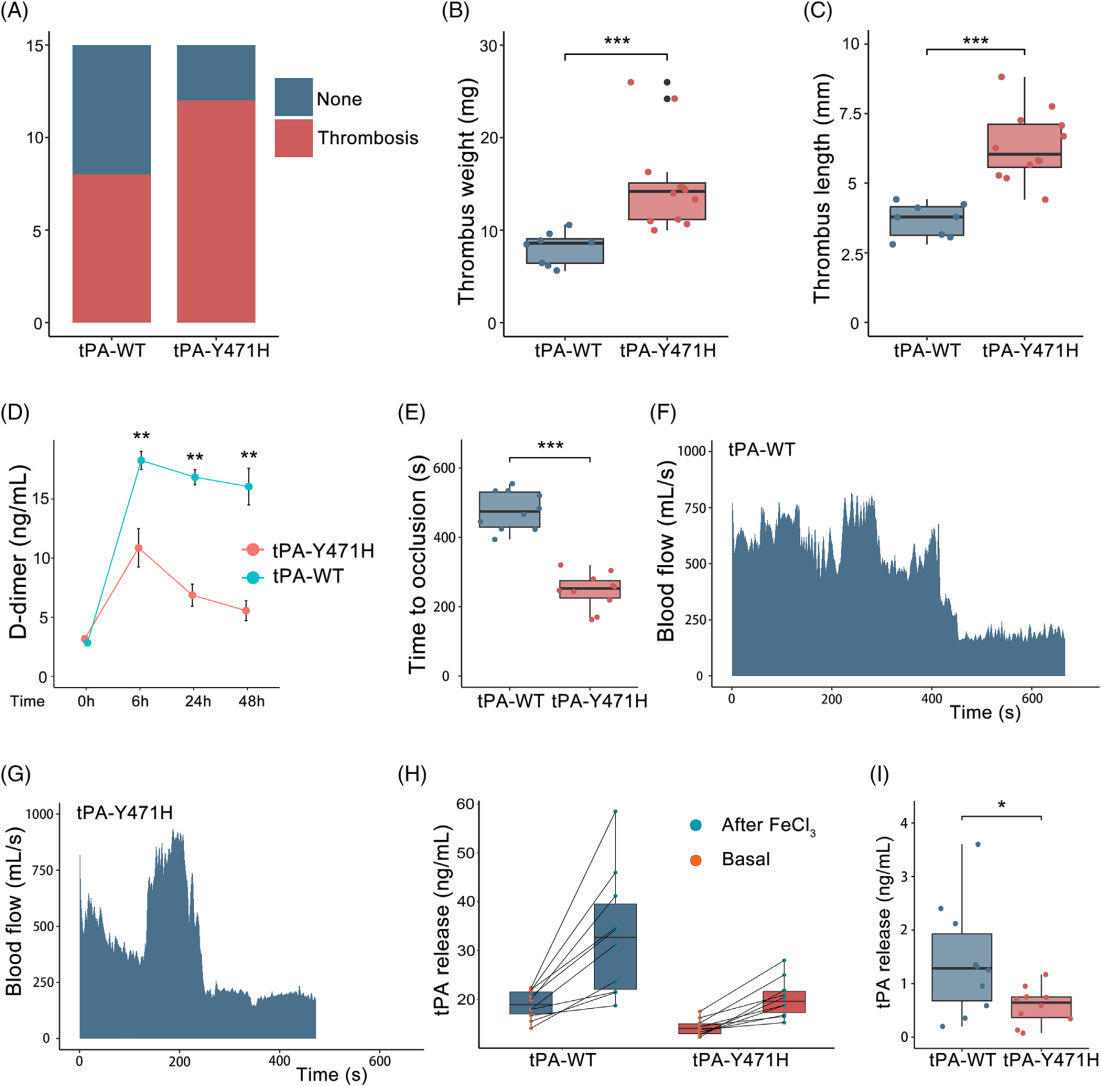
Mutation increases the propensity for thrombosis in *PLAT^H/H^* mice. (A) Venous thrombus formation differed between *PLAT^H/H^* male mice and wild‐type male mice. *N* = 15 mice per group. (B and C) Comparison of average thrombus weight and length between the two groups. Data are presented as median and interquartile range (IQR) where appropriate. An unpaired *t*‐test was used for statistical analysis. ^*^ *p* < 0.05; ^**^ *p* < 0.01; ^***^ *p* < 0.001; ns: not significant. (D) Changes in D‐dimer concentration in plasma of mice before inferior vena cava (IVC) stenosis and 12, 24, and 48 h after IVC stenosis. *N* = 5 male mice per group. (E and G) Time to occlusive carotid arterial thrombosis induced by 10% FeCl_3_ injury. *N* = 10 mice per group. Data are presented as median and IQR where appropriate. (H and I) Plasma tissue‐type plasminogen activator (tPA) concentration 1 week before (basal) and 20 min after FeCl_3_‐induced carotid artery thrombosis; tPA release was calculated by subtracting the basal value from the after‐FeCl_3_ value for each mouse. *N* = 10 mice per group. Data are presented as median and IQR where appropriate.

We used both an inferior vena cava (IVC) restriction model and carotid artery thrombosis model to investigate the effect of tPA mutation on thrombosis in mice. For males, venous thrombus formation was observed in 12/15 (80%) mice in *PLAT^H/H^* male mice and in 8/15 (53%) mice in wild‐type mice (Figure 5A). Compared with the wild‐type male mice, *PLAT^H/H^* male mice showed significantly increased thrombus weight (14.78 ± 4.36 mg vs. 7.7 ± 1.70 mg, *p* < 0.001), and increased thrombus length (6.33 ± 1.20 mm vs. 3.67 ± 0.60 mm, *p* < 0.001) (Figure 5B,C). The results showed that there was a transient increase in DDI concentration in plasma of mice after IVC stenosis (Figure 5D). Compared with the wild‐type male mice, the DDI concentration in the plasma of *PLAT^H/H^* male mice was significantly decreased 12 h after surgery (10.86 ± 3.62 ng/mL vs. 18.26 ± 1.71 ng/mL, *p* = 0.0059), suggesting that the mutation reduced the fibrinolytic ability of mice. And the results suggested that the mutation increased the tendency of venous thrombosis in mice, regardless of sex (Figure S1). In addition, occlusive carotid artery thrombosis induced by FeCl_3_ was shortened in *PLAT^H/H^* mice compared with wild‐type mice (477.8 ± 56.00 s vs. 246.6 ± 51.38 s, *p* < 0.001) (Figure 5E–G). tPA could be rapidly released locally by vascular endothelial cells in response to injury, and we compared the concentration of tPA in the plasma of mice before and 20 min after FeCl_3_‐induced carotid injury. And we found that the relative increase after FeCl_3_ was significantly higher in wild‐type mice than in *PLAT^H/H^* mice (14.15 ± 10.42 ng/mL vs. 5.91 ± 3.27 ng/mL, *p* = 0.0283) (Figure 5H,I), which might be the reason for affecting postinjury fibrinolysis. These data suggested that the mutation increased the propensity for thrombosis in *PLAT^H/H^* mice. Meanwhile, the tail bleeding time after vascular injury in *PLAT^H/H^* mice was significantly shorter than that of wild‐type mice (257 ± 61.29 s vs. 165 ± 27.99 s, *p* < 0.001) (Figure S2).

### 2.8 The mutant tPA had a shorter half‐life

**FIGURE 6:**
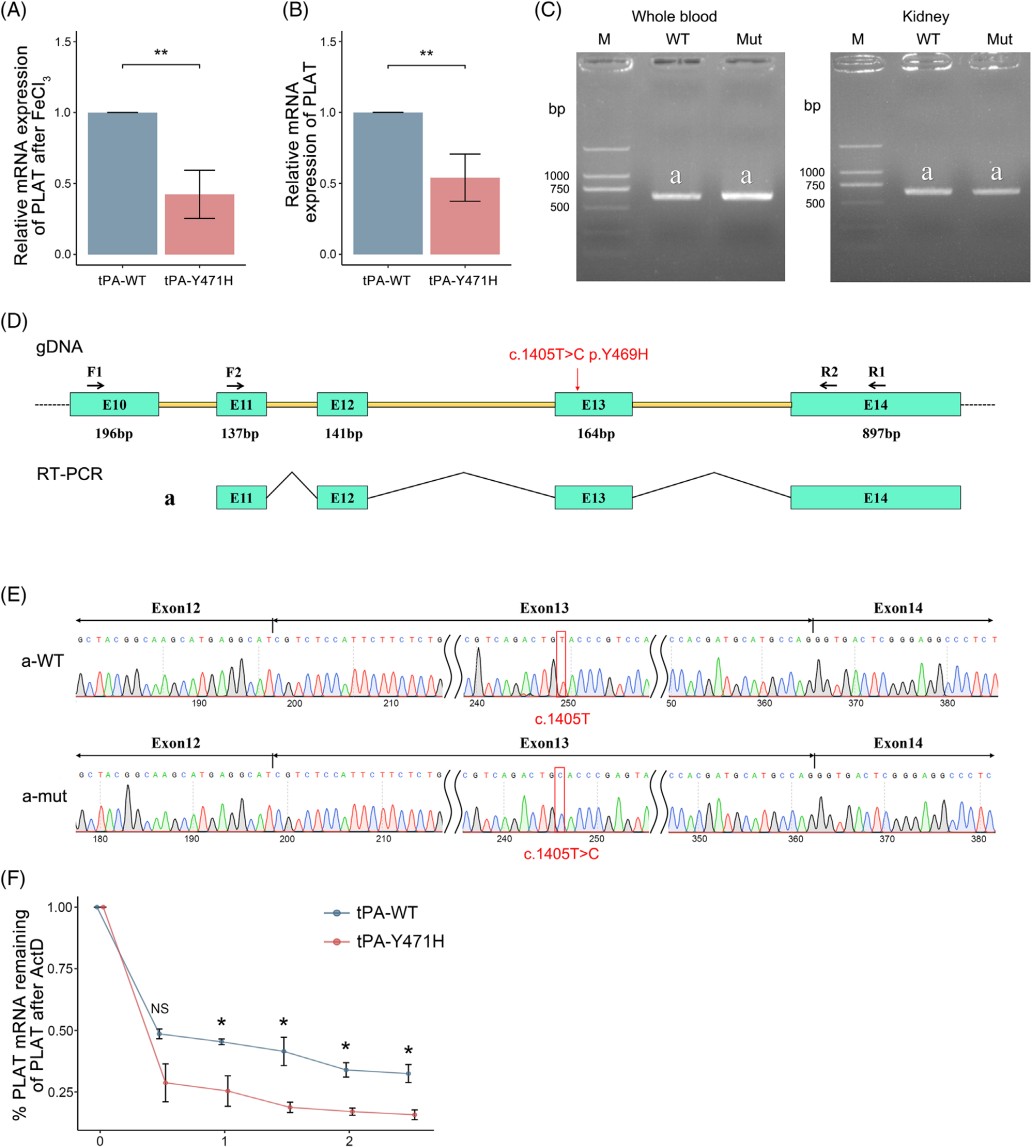
The mutant tissue‐type plasminogen activator (tPA) had a shorter half‐life. (A) *PLAT* mRNA in carotid arterial lysates normalized to 18S and expressed as relative to the value in wild‐type mice. Mice were randomly selected from 20 min after FeCl_3_‐induced injury. *N* = 5 mice per group. Bars represent the group mean ± standard deviation (SD). (B) As described above, the mice were not given any intervention. *N* = 5 mice per group. Bars represent the group mean ± SD. An unpaired *t*‐test was used for statistical analysis. ^*^ *p* < 0.05; ^**^ *p* < 0.01; ^***^ *p* < 0.001; ns: not significant. (C) Agarose gel electrophoresis for the polymerase chain reaction (PCR) products in peripheral blood and kidney (b and a). The results all showed a single band, and the band size was 626 bp as expected. (D) Schematic diagram of primer design and splicing model. The splicing mode of band a is exon 12 (141 bp)–exon 13 (164 bp)–exon 14 (897 bp). The red arrow indicates the location of the mutation. The letter E stands for exon, letter F stands for forward primer, and R stands for reverse primer. (E) Sanger sequencing results of band a in (C). (F) The results of *PLAT* mRNA remaining percentage analysis in sample. *N* = 5 mice per group. The means ± SD (*y*‐axis) are shown over time (*x*‐axis). ^*^ and ns on time denote differences in tissue plasminogen activator with a p.Tyr471His (tPA‐Y471H) from wild‐type tissue plasminogen activator (tPA‐WT).

tPA stored in endothelial granules can be rapidly released when endothelial cells get injured to prevent excessive fibrin deposition and thrombosis. We sought to determine whether tPA secreted by vascular endothelium would be affected by the mutation. Carotid artery lysates from *PLAT^H/H^* mice and wild‐type mice were used to detect *PLAT* expression at mRNA level. The results showed that the mRNA level of *PLAT* was significantly decreased in *PLAT^H/H^* mice (42.39 ± 14.87%) compared with wild‐type mice (100%, *p* = 0.0016) at 20 min after FeCl_3_‐induced carotid injury (Figure 6A). For mice without any intervention, the mRNA expression of *PLAT* was also significantly lower in *PLAT^H/H^* mice (54.03 ± 14.57%) than in wild‐type mice (100%, *p* = 0.0035) (Figure 6B). Since this mutation was located in the *PLAT* coding sequence, we considered that the mutation might affect the post‐transcriptional level of tPA. According to the in vivo mRNA splicing assay, the mutation does not affect normal splicing, and no other ectopic transcripts of *PLAT* were detected in *PLAT^H/H^* mice (Figure 6C–E). Meanwhile, mRNA half‐life assay was used to explore whether the mutation affected the stability of the mRNA of tPA. After treatment of endothelial cells with actinomycin D (ActD), we found that the mRNA stability of tPA in *PLAT^H/H^* mice was significantly decreased compared with that in wild‐type mice (Figure 6F); that is, the half‐life of tPA was shortened, which in turn affected fibrinolysis.

## 3 DISCUSSION

As a widely used thrombolytic drug in clinic, tPA is mainly used in the treatment of acute myocardial infarction, pulmonary embolism, ischemic stroke, deep vein thrombosis, and peripheral arterial occlusion to recanalize blocked blood vessels.^22^, ^23^, ^24^ Current studies on tPA are mainly focused on fibrinolytic function and thrombolysis drug optimization, and few reports have linked tPA with thrombophilia. Here, we report a newly identified rare pathogenic tPA mutation associated with venous thrombosis. The proband developed multiple deep venous thrombosis without obvious inducement. Gene sequencing results showed that the proband had homozygous point mutation c.1411T>C (p.Y471H) in the *PLAT* gene, and it was found that the proband's tPA antigen concentration and activity decreased to 73.2% and 54.8% of those in healthy subjects, respectively. In vitro studies showed that the fibrinolytic activity of tPA‐Y471H was significantly decreased compared with that of tPA‐WT.

tPA protein has five domains, including one protease domain located in the C‐terminal light chain and one finger domain, one epidermal growth factor domain and two kringles domains located in the N‐terminal heavy chain.^25^ Plasminogen activation is positively regulated by the ternary TPA–plasminogen–fibrin complex. tPA interacts with the protease domain of plasminogen via the kringle 2 domain, dependent on conformational changes induced by its finger domain.^7^, ^26^ The kringles 1, 4, and 5 domains of plasminogen have been reported as the major mediators of the plasminogen/fibrin interaction.^27^ The exact mechanism by which the tPA/plasminogen interaction stabilizes the ternary complex remains uncertain.

The mutation site (p.Y471H) reported here is located in the protease domain of tPA, which has the integrated catalytic function of converting plasminogen into plasmin and also the binding site of PAI‐1.^28^ Therefore, we studied the effect of this mutation on the binding of the tPA protease domain to plasminogen and PAI‐1. Molecular docking results showed that the mutation changed the spatial structure of tPA, resulting in conformational dynamics that affect the binding affinity of the tPA protease domain with plasminogen and PAI‐1, resulting in reduced hydrogen‐bonding interaction, reduced free binding energy and weakened affinity. It is noteworthy that the protease domain of tPA‐WT interacts with the kringle 1 and kringle 4 domains of plasminogen, while tPA‐Y471H only interacts with the kringle 1 domain of plasminogen. SPR results also showed that compared with tPA‐WT, the affinity between tPA‐Y471H and PAI‐1 was slightly decreased, while the affinity between tPA‐Y471H and plasminogen was significantly decreased, with a difference of one order of magnitude. We speculated that this mutation affected the binding form of tPA and plasminogen, affected the stability of the ternary complex, and thus affected the effective activation of fibrin by plasminogen. This may be a key regulatory step in the involvement of the tPA protease domain in plasminogen activation, and an important reason for the decreased fibrinolytic function of tPA variant.

The mouse and human *PLAT* genes share 79% identity, and the homology between the mouse and human tPA proteins is 80%. The tyrosine at position 471 of the human tPA protein corresponds to the mouse tyrosine at position 469. The results showed that changes in tPA antigen level and enzyme activity in *PLAT^H/H^* mice were consistent with those in the proband (antigen concentration decreased to 74.7% of that in wild‐type mice, activity decreased to 56.8% of that in wild‐type mice). And we found that the plasma levels of plasminogen, PAI‐1, FIB, DDI, and FDP in *PLAT^H/H^* mice have no significant difference from those in wild‐type mice. The relative concentrations of plasma tPA and its inhibitor PAI‐1 largely determine the fibrinolytic function of plasma.^29^ Through the mouse model of inferior vena cava stenosis, we found that *PLAT^H/H^* mice had an increased propensity for thrombosis, and the venous thrombus formed were heavier and longer than that of wild‐type mice. These combined data demonstrate that *PLAT^H/H^* mice have decreased fibrinolysis.

Low plasma tPA activity is considered an independent predictor of cardiovascular disease in humans.^30^, ^31^, ^32^ Vascular endothelial cell injury promotes the local rapid release of tPA.^33^, ^34^ Our results showed that *PLAT^H/H^* mice had significantly reduced tail bleeding time as well as significantly shorter occlusion time of thrombus formation in the model of carotid artery injured by FeCl_3_, which might be related to lower tPA concentrations released from the endothelium after injury than in wild‐type mice. Notably, *PLAT* mRNA expression in carotid endothelial cells was significantly lower in *PLAT^H/H^* mice than in wild‐type mice both before and after FeCl_3_‐induced carotid injury. Since the site of the mutation is in the *PLAT* coding sequence, which is not a cis‐acting element, it is more likely that the mutation may affect the post‐transcription level than the transcription level. An in vivo splicing assay showed that the mutation did not affect the normal splicing of *PLAT*, and no other ectopic transcripts of *PLAT* were found in *PLAT^H/H^* mice. We treated endothelial cells with transcription inhibitor ActD to explore the effect of mutation on post‐transcription level.^35^, ^36^ The data showed that tPA mutation resulted in decreased *PLAT* mRNA stability in endothelial cells; that is, the half‐life of mutant tPA was shorter than that of tPA‐WT. Using as a “gold standard” for thrombolytic drugs, tPA has a very short half‐life of about 3 min, and how to prolong the half‐life of tPA is the latest research direction of thrombolysis.^37^ This may also be an important reason for the fibrinolysis dysfunction. rtPA may be considered for the treatment of thromboembolic events in patients with this homozygous point mutation.

Currently, there are insufficient data to verify the association of mutations in the *PLAT* gene with venous or arterial thrombosis.^38^, ^39^ According to the prediction, the mutation c.1411T>C (p.Y471H) in *PLAT* is predicted to be probably harmful (http://genetics.bwh.harvard.edu/pph2/), which is consistent with our clinical findings and research results. However, it has also been reported that the heterozygous point mutation c.1411T>C in *PLAT* is likely to be a non‐pathogenic benign mutation (http://www.gnomad‐sg.org/), which may be the reason why the parents and daughter of the proband did not have venous thromboembolism events despite carrying heterozygous point mutation c.1411T>C in *PLAT*.

Our work also has some limitations. More clinical data are needed to further explore the incidence of this rare homozygous point mutation. Further research should be done to explore the mechanism and significance of the tPA protease domain involved in plasminogen activation. Our work has not ruled out the possibility that the mutation affects the tPA transcription level, and the reason why this mutation affects the post‐transcription level of tPA and shortens the half‐life of tPA deserves further study.

## 4 CONCLUSIONS

In summary, our study reported a newly homozygous point mutation (c.1411T>C) located in the protease domain of tPA, investigated the mechanism of tPA functional deficiencies caused by this mutation, and demonstrated that this mutation was associated with an increased thrombotic tendency, which provides new insights and ideas for the diagnosis and treatment of patients with thromboembolic diseases in clinic. Meanwhile, our data suggested a novel role for the interaction between the protease domain of tPA and the kringle 4 domain of plasminogen in efficient plasminogen activation, providing a unique target for controlling plasmin generation.

## 5 MATERIALS AND METHODS

### 5.1 Patients and samples

The proband was admitted because of deep vein thrombosis of the lower extremities. Peripheral blood samples of the proband were collected for whole‐genome sequencing and clinical data testing. Saliva samples of the proband's parents and daughter were obtained for gene sequencing. The protocol was approved by the Ethics Committee of the Union Hospital affiliated with Huazhong University of Science and Technology (number: [2022] 0157). We obtained informed written consent from all subjects prior to their inclusion.

### 5.2 Synthesis of tPA‐WT and tPA‐Tyr471His

The full‐length tPA‐WT and tPA‐Tyr471His (tPA‐Y471H) were obtained using baculovirus expression system from a supplier (CUSABIO). The synthesized and purified tPA‐WT and tPA‐Y471H were verified by Western blotting, as shown in Figure S3.

### 5.3 Origin of point mutant mice

The point mutation *PLAT^H/H^* mice carrying the *PLAT* Y469H mutation (corresponding to human Y471H, C57BL/6N) were generated by supplier (Cyagen Biosciences) using CRISPR/Cas‐mediated genome engineering. The mouse *PLAT* gene (GenBank accession number: NM_008872.3) is located on mouse chromosome 8, and the Y469 is located on exon 13. Therefore, we selected exon 13 as target site. The Y469H (TAC to CAC) mutation sites in donor oligo were introduced into exon 13 by homology‐directed repair. A silent mutation (TCC to AGT) was also introduced to prevent the binding and re‐cutting of the sequence by gRNA after homology‐directed repair. The pups were genotyped by polymerase chain reaction followed by sequence analysis.

### 5.4 Surface plasmon resonance

The interaction of tPA (tPA‐WT or tPA‐Y471H) with plasminogen (Sigma–Aldrich) and PAI‐1 (Sino Biological) was analyzed in real time by SPR using an Open SPR (Nicoya).^40^ Briefly, the ligand (tPA‐WT or tPA‐ Y471H, 20 μg/mL) was immobilized onto COOH‐sensor chips (Nicoya) by EDC/NHS chemistry. Analytes serially diluted in buffer (140 mM NaCl,10 mM N‐2‐hydroxyethylpiperazine‐N9‐2‐ethanesulfoic acid, pH 7.4) were then injected and captured at a flow rate of 20 μL/min for 240 s. Kinetic parameters for the binding reactions were calculated and analyzed using Trace Drawer software (Ridgeview Instruments AB).

### 5.5 Molecular docking

The docking of tPA protease domain to plasminogen and PAI‐1 was performed on the network script HDOCK server (http://hdock.phys.hust.edu.cn/),^41^ respectively. In brief, the 3D structures of tPA (ID: 1RTF), plasminogen (ID: 4DUR), and PAI‐1 (ID: 3Q02) retrieved from Protein Data Bank (http://rcsb.org/) were uploaded to the HDOCK server for molecular docking.^42^, ^43^ The scoring function was used to perform rigid docking of the two proteins, and the docking yielded 100 conformations. We selected the protein binding model with the lowest docking energy score and used PyMOL 12.1 to display the 3D graphics of this model.^44^

### 5.6 Western blot for plasmin generation

The ability of tPA to cleave plasmin to plasmin was evaluated by incubating plasminogen (75 nmol/L) with tPA (200 nmol/L) in tris‐buffered saline (TBS, Servicebio) added FIB (235 nmol/L, Sigma–Aldrich), CaCl_2_ (2.5 mM, Sigma–Aldrich), and thrombin (2 U/mL, HYPHEN BioMed, Neuville‐sur‐Oise) at 37°C.^45^ The total reaction volume was 200 μL. A total of 20 μL was removed at 2‐min time intervals and frozen before Western blotting, and densitometry analysis was performed using an imaging analyzer (Bio‐Rad) and the ImageJ software. The details of antibodies used can be found in Table S2.

### 5.7 Immunofluorescence imaging

Fibrin clots were formed from 100 μL of plasminogen‐depleted plasma (Affinity Biosciences), 10 μL of Alexa Fluor 594‐labeled FIB (1 mg/mL, Thermo Fisher), 5 μL of CaCl_2_ (100 mM), and 10 μL of thrombin (5 IU/mL) for 15 min at 37°C in sterile slides.^40^, ^46^, ^47^ Lysis buffer containing tPA (10 μg/mL) and plasminogen (1 mg/mL) was dropped on the edge of the clot. Fluorescence was observed in real time with a Leica DMi8 inverted fluorescence microscope (Leica Microsystems). Images of the liquid–fibrin interface were taken continuously with the same exposure conditions using a 20×/0.5 objective lens. Several time points (0, 5, and 10 min) were selected to measure the distance of the lysis peak from the set fixed point. The velocities of the cleavage front at six different positions along the cleavage front were recorded, and the average total cleavage rate (μm/min) was calculated.

### 5.8 Determination of fibrinolytic activity of tPA

Agar plates were utilized to detect the fibrinolytic activity of tPA, and 150 mg of low‐melting agar was dissolved in 5 mL Tris–HCl buffer (pH 7.4) by microwave heating. After cooling to 37°C, 5 mL of FIB solution (1 mg/mL) and 500 μL of thrombin (5 IU/mL) were added into it and the mixture was stirred for 30 s. The mixed solution was packed in a 9 cm Petri dish and incubated at 37°C for 2 h to form a solidified fibrin clot plate. Three wells (I.D. 3 mm) were created as sample reservoir. To each well, 3 μL of plasminogen (1 mg/mL) and 2 μL of phosphate‐buffered saline (Gibco) as control, tPA‐WT solution (100 μg/mL), and tPA‐Y471H solution (100 μg/mL) were added and incubated at 37°C overnight for complete fibrinolysis. Fibrinolytic activity was assessed by calculating the area of the lytic region around the pore.^48^

### 5.9 mRNA half‐life assay

Carotid endothelial cells were isolated from wild‐type mice and *PLAT^H/H^* mice as described.^49^, ^50^ Endothelial cells were counted and seeded into 12‐well plates at 1 × 10^6^ cells per well and cultured at 37°C under 5% CO_2_ in Dulbecco's modified Eagle's medium containing 10% fetal bovine serum. After 6 h of culture, ActD (10 μg/mL) was added to the culture medium, followed by incubation for 15 min, 30 min, 1 h, 2 h, or 3 h. Real‐time reverse‐transcription polymerase chain reaction (RT‐PCR) was performed to measure the levels of tPA mRNA. 18S RNA was detected as control and the sequence of primers used is shown in Table S2.^35^

### 5.10 Inferior vena cava stenosis model

All experiments involving animals in this research followed the ethical standards set by the Institutional Animal Care and Use Committee of Tongji Medical College, Huazhong University of Science and Technology (number: [2021] 3107). Thirty *PLAT^H/H^* mice (15 males and 15 females, 20–24 g, 8 weeks old) were randomly selected to establish the IVC mouse model.^51^ Thirty wild‐type mice (C57BL/6N) of the same age and body weight were used as controls. The mice were continuously anesthetized with isoflurane–oxygen mixture and placed in supine position to expose the IVC after a midline laparotomy. The remaining branches of the IVC were ligated below the renal vein and above the iliac vein. The flow of the IVC was limited by ligation with 7‐0 polypropylene suture above a 31 G diameter needle. The needle was carefully removed and the peritoneum and skin were sutured. At 12, 24, and 48 h after surgery, five male mice were randomly selected to collect 200 μL of blood from the inner canthus, and the concentration of DDI in the plasma of the mice was detected by Mouse D‐Dimer ELISA Kit (Elabscience). The details of ELISA kit can be found in Table S1. The DDI concentration measured from the plasma of five randomly selected male mice was used as the base value. Forty‐eight hours after surgery, the thrombus formed below the sutures in the IVC was removed. After drying the thrombus, the weight of the thrombus was weighed by a precision balance, and the length of the thrombus was measured with a Vernier caliper.

### 5.11 Carotid artery thrombosis model and tPA release

Twenty microliters of plasma was collected via tail bleed 1 week before the carotid artery thrombosis model as a base value. The carotid artery thrombosis was the result of FeCl_3_‐induced injury as described previously.^52^, ^53^ The detailed protocol is provided in the Supporting Information.

### 5.12 Statistical analysis

The data are presented as mean ± standard deviation of the mean. Student's *t*‐test was used to identify significant differences in comparisons between any two groups. A two‐tailed *p* < 0.05 was considered statistically significant. Statistical analyses were conducted using GraphPad Prism version 8 and R software.

## AUTHOR CONTRIBUTIONS

L.V.T., Y.H., and Y.Y.T. conceptualized and designed the study. Y.Y.T. and Z.P.C. recruited the patients. Y.Y.T., J.W.M., Y.Z.F., Y.Z., T.T.W., and Y.Q.X. performed experiments. Y.Y.T. and C.G.G. completed statistical analyses and created the figures. Y.Y.T. wrote the original manuscript. L.V.T. revised the manuscript. All authors have read and approved the final manuscript.

## CONFLICT OF INTEREST STATEMENT

The authors declare they have no conflicts of interest.

## ETHICS STATEMENT

The studies involving human participants were reviewed and approved by the Ethics Committee of the Union Hospital affiliated to Huazhong University of Science and Technology (number: [2022] 0157). Informed written consent was obtained before the inclusion of subjects in accordance with the Declaration of Helsinki. All experiments involving animals in this research followed the ethical standards set by the Institutional Animal Care and Use Committee of Tongji Medical College, Huazhong University of Science and Technology (number: [2021] 3107).

## Supporting information

Supporting Information

## Data Availability

All the presented information in this article is accessible by contacting the corresponding author.
