# Supplementary material for: Tissue‐type plasminogen activator (tPA) homozygous Tyr471His mutation associates with thromboembolic disease
Source: MedComm (2020). 2023 Oct 5;4(5):e392. doi: 10.1002/mco2.392 (PMC10556205; doi:10.1002/mco2.392)
Supplement: Supplementary file 1 — Supporting Information [file MCO2-4-e392-s001.docx]

**Tissue-type plasminogen activator** (**tPA)** **homozygous Tyr471His mutation associates with** **thromboembolic** **disease**

**Running title：tPA Y471H mutation causes** **thromboembolism**

Yanyi Tao^1^, Jiewen Ma^1^, Yuanzheng Feng^1^, Chenggang Gao^2^, Tingting Wu^1^, Yunqing Xia^1^, Zhipeng Cheng^1^, Yi Zhang^1^, Tingting Liu^1^, Yu Hu^1*^, Liang V. Tang^1,*^

^1^^.^ Institute of Hematology, Union Hospital, Tongji Medical College, Huazhong University of Science and Technology; Wuhan 430022, China

^2.^ Department of Critical Care Medicine, Union Hospital, Tongji Medical College, Huazhong University of Science and Technology, Wuhan, China

***Corresponding author.** (lancet_tang@hust.edu.cn) or ([dr_huyu@126.com](mailto:dr_huyu@126.com))

**Supplemental Materials**

**Materials and Methods**

**Mouse tail bleeding assay**

Mice were anesthetized with isoflurane and positioned horizontally on a platform that allowed the tail to drop about 2 cm from the top of the platform. A segment of tail on the distal tail tip was transected with a no. 21 surgical scalpel to induce wounds about 2 mm in diameter. Blood that continued to flow from the cut during the 10-second interval was allowed to fall on the Whatman filter paper (Whatman International Ltd, Maidstone, UK) at the same point. The time to stable bleeding stop was defined as the interval between tail incision and bleeding stop, with no signs of rebleeding within 60 seconds.

**In vivo mRNA splicing assay**

Peripheral blood and vascular-rich kidney tissue samples of *PLAT^H/H^* mice and wild-type mice were collected, mRNA was extracted and cDNA synthesized by reverse transcription. We constructed two pairs of nested primers for two rounds of PCR amplification to facilitate better detection of other splicing products that may have lower expression. The primer sequences are provided in Table S3. The size of products was detected by agarose gel and the products were subjected to Sanger sequencing.

**Fibrin clot lysis**

Clot lysis was performed by measuring the turbidity of plasma in which thrombin induced clot went through tPA mediating fibrinolysis. Blood sample was collected from healthy individual and 0.129 M sodium citrate was used as anticoagulant, and plasma was collected after centrifugation (15 min, 2300g). Human thrombin (10ul at 5 UI/ml), CaCl2 (5ul at 100 mM) and 25 µl citrated plasma were incubated in 55 µl HEPES buffer (20 mM HEPES, pH 7.4) for 15 min at 37℃. After adding tPA (5ul at 20 nM) into it, the mixture’s turbidity was kinetically measured at 405 nm in a PerkinElmer Enspire reader. The reading was taken every minute for 2h at 37℃. The clot lysis time was defined as the time interval between the maximal turbidity and the midpoint of the turbid-to-clear transition that characterized the lysis.

**Plasma collection and analyses**

Blood sampled from mice eyes in 10% volume of sodium citrate (3.8%, weight-to-volume ratio) was centrifuged for 15 minutes at 2300g, and plasma was carefully collected from the supernatant fraction. Plasma samples were parceled out and stored at −80°C until analyses. Total antigen levels of tPA, plasminogen, PAI-1 in plasma were measured by enzyme-linked immunosorbent assay (ELISA) kits, according to manufacturer’s instructions (catalog numbers are listed in Supplementary Table S1). Fibrinogen (FIB), D-dimer (DDI) and fibrin degradation products (FDPs) in plasma were measured by an automatic coagulator (Sysmex, Kobe, Japan). tPA enzymatic activity in plasma was determined using a commercial Chromogenic Activity Kit (Assaypro, USA).

**Carotid artery thrombosis model and tPA release**

Briefly, the right common carotid artery was exposed and dried after the mice were anesthetized, then a filter paper soaked in 10% FeCl_3_ was applied to the artery for 2 minutes, followed by washing with warm saline and blood flow was continuously monitored by Laser Doppler Flowmetry (Moor Instruments, UK). The occlusion time was defined as the time interval between the end of FeCl_3_ damage and stable occlusion of the artery, with no blood flow for 3 minutes. Plasma was collected 20 minutes after FeCl_3_-induced carotid injury. Basal and post-FeCl_3_ plasma tPA antigen levels were measured on the same ELISA plate. The tPA release data were calculated by subtracting the post-FeCl_3_ value from the basal value for each mouse.

**Supplemental Figure Legends**


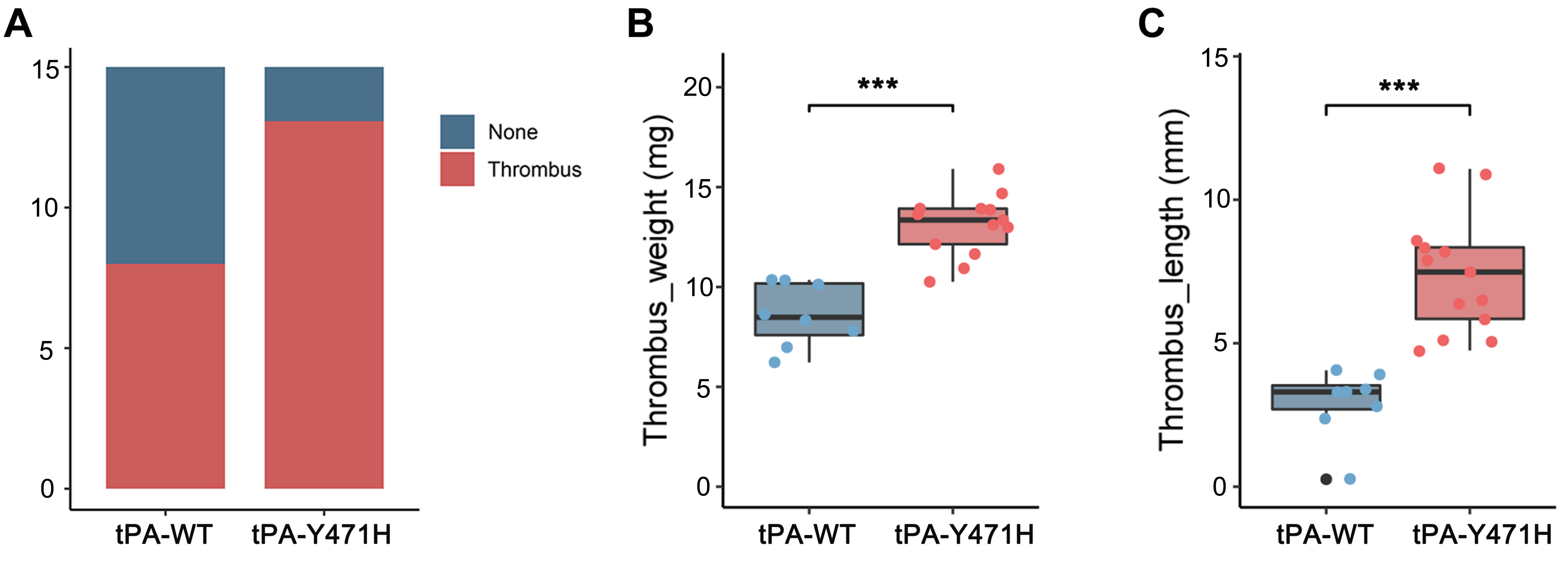


**Figure S1. Effect of tPA mutation on the propensity for venous thrombosis in female mice.**

(A) Venous thrombus formation differed between *PLAT^H/H^* female mice and wild-type female mice. N = 15 mice per group. (B, C) Comparison of average thrombus weight and length between the two groups. Data are presented as median and interquartile range (IQR) where appropriate. Unpaired t-test was used for statistical analysis. *P < 0.05; **P < 0.01; ***P < 0.001. ns: not significant.


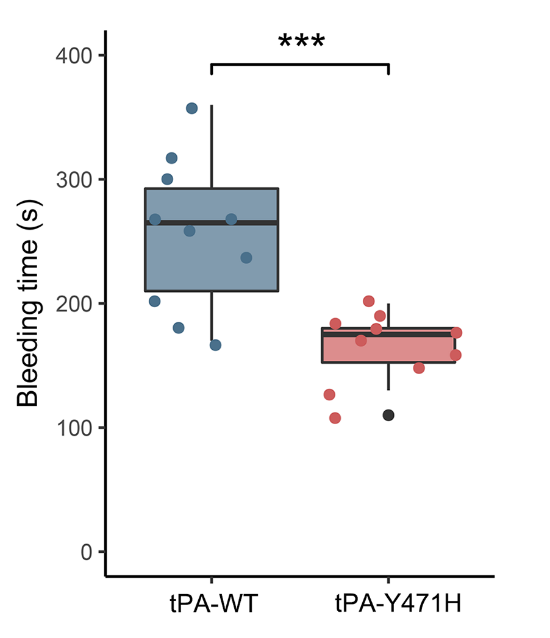


**Figure S2. The tail bleeding time after vascular injury in *PLAT^H/H^* mice and the wild-type mice.**

Tail bleeding time was determined as the time taken for the cessation of bleeding after transection. N = 10 mice per group. Data are presented as median and IQR where appropriate.

**
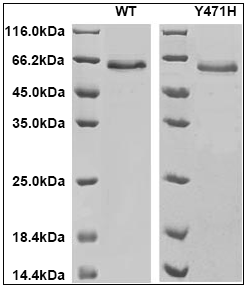
**

**Figure S3. The verification of synthesized** **tPA-WT and tPA-Y471H.**

The synthesized tPA-WT and tPA-Y471H showed a band size at 62.9 kDa.

**Supplemental Tables**

**Table S1. Plasma levels of fibrinolytic factors (n = 8 mice per group).**

| Fibrinolytic factors | tPA-WT | tPA-Y471H | P value |
| --- | --- | --- | --- |
| tPA_antigen, ng/mL | 18.89 ± 2.55 | 14.11 ± 2.33 | 0.0016 |
| tPA_activity, UI/mL | 120.89 ± 12.59 | 68.64 ± 5.35 | <0.0001 |
| Plasminogen, ng/mL | 1164.82 ± 203.42 | 1114.63 ± 186.44 | 0.6151 |
| PAI-1, ng/mL | 2.96 ± 0.61 | 3.12 ± 0.94 | 0.6350 |
| FIB, g/L | 2.88 ± 0.60 | 2.97 ± 0.42 | 0.7448 |
| DDI, ug/mL | 0.22 ± 0.05 | 0.21 ± 0.03 | 0.8040 |
| FDP, ug/mL | 2.58 ± 0.99 | 2.14 ± 0.52 | 0.2868 |

Values are presented with mean ± SD. Abbreviations: tPA, tissue plasminogen activator; tPA-WT, wild type tissue plasminogen activator; tPA-Y471H, tissue plasminogen activator with a p.Tyr471His; PAI-1, plasminogen activators inhibitor-1; FIB, fibrinogen; DDI, D-dimer; FDP, fibrin degradation products.

**Table S2. ELISA kits and antibodies.**

| MATERIALS | SOURCE | IDENTIFIER |
| --- | --- | --- |
| Human tPA antigen ELISA Kit | Assaypro | ET1001-1 |
| Mouse total tPA antigen ELISA Kit | Molecular Innovations | MTPAKT-TOT |
| Mouse Plasminogen ELISA Kit | CUSABIO | CSB-EL018188MO |
| Mouse Plasminogen Activator Inhibitor 1 ELISA Kit | Elabscience | E-EL-M3041 |
| tPA Chromogenic Activity Kit | Assaypro | CT1001 |
| Rabbit anti-Plasminogen | Abcam | ab174285 |
| Goat Anti-Rabbit IgG H&L (HRP) | Abcam | ab205718 |

**Table S3. Primers used for PCR.**

| Gene Symbol | Sequence（5′→3′） |
| --- | --- |
| PLAT | F: CTGACGTGGGAATACTGTGACAT |
|  | R: GTGTGAGGTGATGTCTGTGTAGA |
| 18S | F: GGCCGTTCTTAGTTGGTGGAGCG |
|  | R: CTGAACGCCACTTGCCCCTC |
| ROUND1 | F: ACATCACCTCACACCCTTGG |
|  | R: GTTGGCATCTCCCTGTCATC |
| ROUND2 | F: CCGGAGAGGAAGAACAGACA |
|  | R: CCTTTTAGGCGCATCTTCTG |
